# Bovine Papillomavirus Type 1 Infection in an Equine Congenital Papilloma

**DOI:** 10.3390/pathogens12081059

**Published:** 2023-08-18

**Authors:** Raffaella Maggi, Livia De Paolis, Daria De Santis, Valerio Gaetano Vellone, Chiara Grazia De Ciucis, Floriana Fruscione, Katia Mazzocco, Alessandro Ghelardi, Giuseppe Marruchella, Elisabetta Razzuoli

**Affiliations:** 1 Veterinary Practitioner, Via Cassia 829, 00189 Rome, Italy; 2 Istituto Zooprofilattico Sperimentale del Piemonte, Liguria e Valle d’Aosta, National Reference Center of Veterinary and Comparative Oncology (CEROVEC), Piazza Borgo Pila 29/34, 16129 Genova, Italy; livia.depaolis@izsto.it (L.D.P.); chiaragrazia.deciucis@izsto.it (C.G.D.C.); floriana.fruscione@izsto.it (F.F.); 3 Veterinary Practitioner, Via San Manno 19, 03024 Cepranno, Italy; dariadesantisvet@gmail.com; 4 U.O.C. Anatomia Patologica, IRCCS Istituto Giannina Gaslini, 16147 Genova, Italy; valeriovellone@gaslini.org (V.G.V.); katiamazzocco@gaslini.org (K.M.); 5 Azienda Usl Toscana-Ovest, UOC Ostetricia e Ginecologia, Ospedale Apuane, 54100 Massa, Italy; ghelardi.alessandro@gmail.com; 6 Faculty of Veterinary Medicine, University of Teramo, 64100 Teramo, Italy

**Keywords:** horse, congenital papilloma, bovine papillomaviruses

## Abstract

Papillomas are benign epithelial lesions protruding on the epithelial surfaces as finger-like or warty projections. These lesions are often caused by papillomavirus (PV) infection. Congenital papillomas have been reported in foals. However, to date, no evidence of PV infection has been provided. In the present paper, we describe the main clinical–pathological features of a congenital papilloma observed in a foal. In addition, biomolecular tests demonstrated BPV1 infection in the case under study. Such data stimulate further investigations, even on archived samples, aiming to clarifying the etiology of equine congenital papilloma and the clinical relevance, if any, of BPV1 vertical transmission in horses.

## 1. Introduction

Papillomas are benign epithelial lesions protruding from the epithelial surfaces as finger-like or warty projections [1]. Papillomas are usually caused by papillomaviruses (PVs, fam. *Papillomaviridae*), non-enveloped and host-adapted viruses provided with a circular dsDNA genome [2]. Microscopically, cutaneous papillomas consist of arborizing fibrovascular fronds lined with a thickened epidermis, more prominent within the stratum spinosum. Large, variably shaped keratohyalin granules, and degenerating cells (so called koilocytes) are commonly observed. Moreover, a few intranuclear viral inclusions can be detected throughout the stratum spinosum and granulosum [3,4].

As far as the horse is concerned, three syndromes caused by *Equus caballus papillomaviruses* (EcPVs) have been described:

- classic equine viral papillomatosis via EcPV-1, which usually affects the muzzle and lips of young horses (<3 years of age) and regresses spontaneously within 2–3 months;
- equine genital papillomas caused by EcPV-2, which affect older horses and do not resolve spontaneously;
- equine ear papillomas caused by EcPV3-6, which present with bilateral and symmetric white hyperkeratotic plaques confined to the pinnae, and rarely resolve spontaneously [3].

In addition, equids develop sarcoids after bovine PV (BPV) type 1 and 2 infection, this being among the very few cases of cross-species PV infection reported so far. Sarcoids are locally invasive fibroblastic tumors, and they represent the most common and economically relevant neoplasms in horses [3,5].

Congenital papillomas have also been reported in foals. To date, no evidence of EcPV infection has been provided, suggesting that equine congenital papillomas might be regarded as epidermal hamartomas [6,7]. The present paper describes the main clinical–pathological features of a congenital papilloma recently observed in a foal. The presence of PV infection was also investigated.

## 2. Case Description

**Figure 1:**
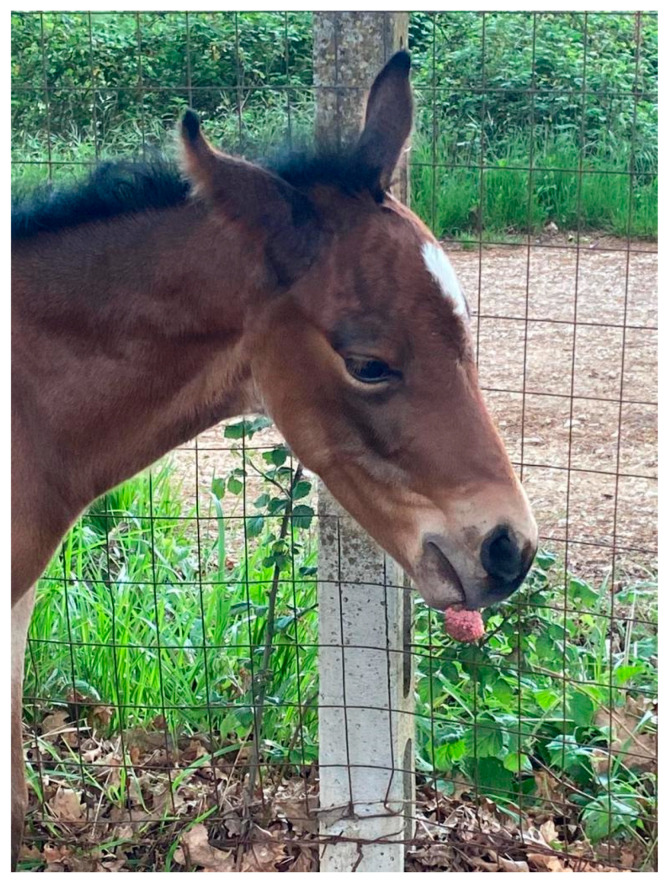
Foal three days after birth. A globoid lesion is clearly seen, attached to the upper lip.

In March 2022, a male, newborn quarter horse foal underwent a routine clinical examination. The foal was born on his due date, and appeared healthy, bright, and alert. However, a spheric, pedunculated, and non-painful mass was observed, which hung from the upper lip and did not prevent suckling (Figure 1).

**Figure 2:**
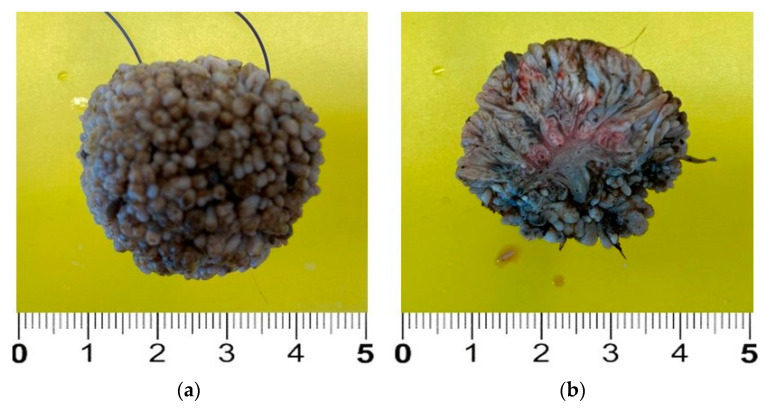
Excised lesion after formalin fixation. The mass is about 3 cm in diameter and shows a verrucous, “truffle-like” surface (**a**). In the cut section, the arborized stromal scaffold can be clearly observed (**b**).

The mass was surgically removed three days later. To achieve this, the foal was drug-free restrained [8], 1 mL of 2% lidocaine chlorydrate was locally injected around the base of the peduncle, and the mass was fully removed and promptly fixed in 10% neutral-buffered formalin (Figure 2). The skin incision healed without any complication, and the stiches were removed 10 days after surgery.

**Figure 3:**
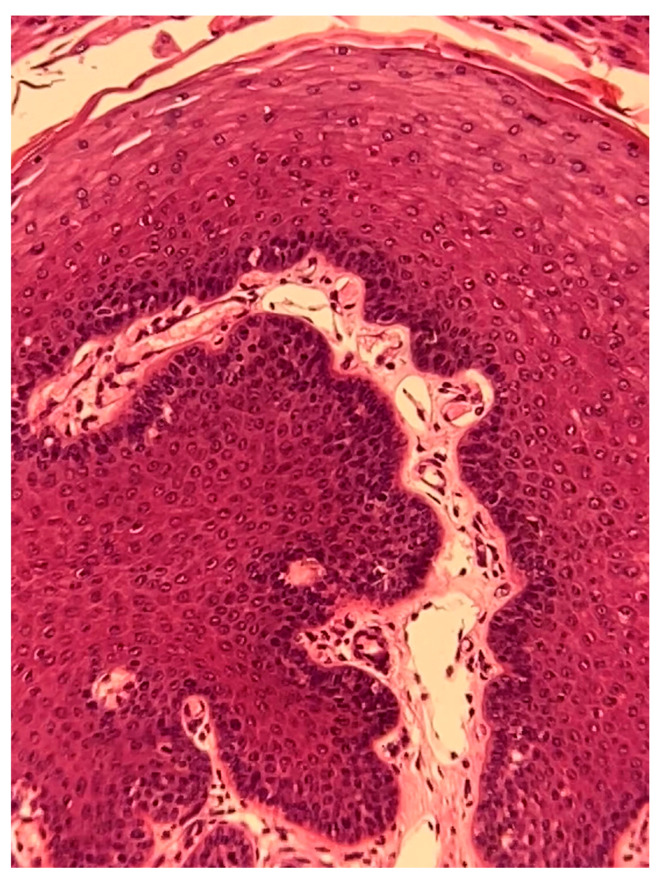
Histopathological examination of the excised lesion. In this finger-like projection, a central fibrovascular backbone is clearly seen, which contains several blood and lymphatic vessels. The stroma is covered by a thickened epidermis, the stratum spinosum being markedly expanded. H and E stain. Final magnification ×200.

**Figure 4:**
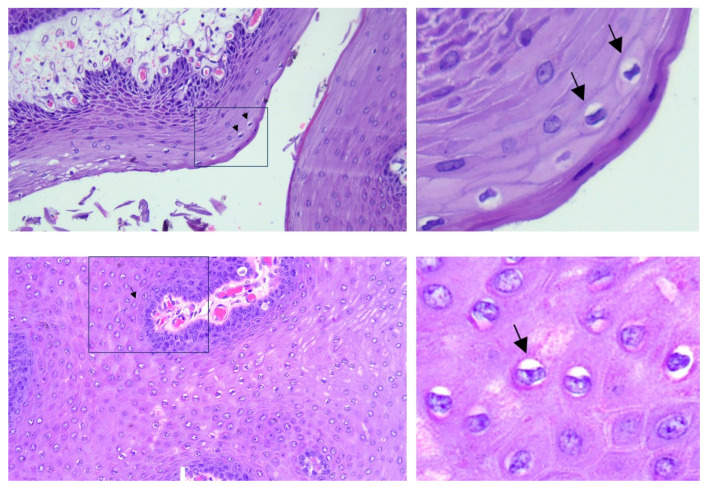
In these sections, evidence of koilocytosis was observed. The right panels reveal a close-up view of the inset. The arrows indicate some selected koilocytes. H and E stain. Final magnification ×200.

Formalin-fixed samples were embedded in paraffin and routinely processed in preparation for histopathological examination (hematoxylin and eosin stain, H and E). Microscopically, the mass consisted of several finger-like fibro-vascular projections, which were covered by a thickened epidermis with prominent acanthosis and orthokeratotic hyperkeratosis (Figure 3). We observed evidence of koilocytosis, keratohyalin granule abnormalities, and a pilosebaceous unit (Figure 4).

Based on the clinical history, gross findings, and histopathological features, the diagnosis of equine congenital papilloma was made.

Total DNA was extracted from three 5 µm thick sections of formalin-fixed and paraffin-embedded (FFPE) samples using the AllPrep DNA FFPE kit (Qiagen, Milan, Italy), according to the manufacturer’s instruction. The DNA concentration was measured using the Qubit 3 fluorimeter (Thermo Fisher Scientific, Waltham, MA, USA). Aiming to investigate the presence of the EcPV2/9/10 and BPV1/2/13 genome, 100 ng of DNA, 200 nM of the probe, and 100 nM of each primer were added to 20 µL of the iTaq Universal Probes Supermix (BioRad, Milan, Italy), in a total volume of 25 µL. Equine beta-2-microglobulin (β2M) was used as the gene reference. Specific primer and probe sequences are reported in Table 1. Internal controls (block blanks, extraction blanks, and positive controls) were used for each analytical session. Real-time PCR was performed using a CFX96 Real-Time System (BioRad, Milan, Italy). A threshold cycle of 38 was set as the cut off for sample positivity. All samples were tested in duplicate.

Real-time PCR demonstrated the presence of the BPV1 genome, while it gave a negative result for all the other PVs herein investigated (see Table 2 and Figure S1 for details).

Thereafter, to evaluate the expression of BPV1-L1 and oncogenes (namely, E5, E6, E7, and L1), total RNA was extracted from three 5 µm thick sections of the FFPE samples using the Maxwell^®^RSC RNA FFPE kit (Promega, Madison, WI, USA), according to the manufacturer’s instructions. RNA concentration was evaluated using the Qubit 3 fluorimeter (Thermo Fisher, Milan, Italy). The reverse transcription (RT) step was performed using the Reliance Select cDNA Synthesis Kit (BioRad, Milan, Italy), adding 100 ng of RNA. Then, 5 μL of 1:3 diluted complementary DNA (cDNA) was added to 20 μL PCR mixture at the final concentration of 1× master mix (iTaq Universal Probes Supermix, Bio-Rad, Irvine, CA, USA), with the following thermal profile: 95 °C for 10′, then 39 cycles of 95 °C for 15′′ and 60 °C for 60′′ in a CFX96™ Real-Time System. To exclude genomic DNA contamination, a digestion using the DNase I RNase free kit (Qiagen, Milan, Italy) was performed. The specific primer set and probes used for testing the expression of BPV1 oncogenes are reported in Table 1. RT-Real-Time PCR demonstrated that BPV1 L1 (Cq = 37.1 ± 0.3), E5 (Cq = 30.1 ± 0.2), E6 (Cq = 34.2 ± 0.2), and E7 (Cq = 33.8 ± 0.3) were expressed at the mRNA level (Table 3).

**Figure 5:**
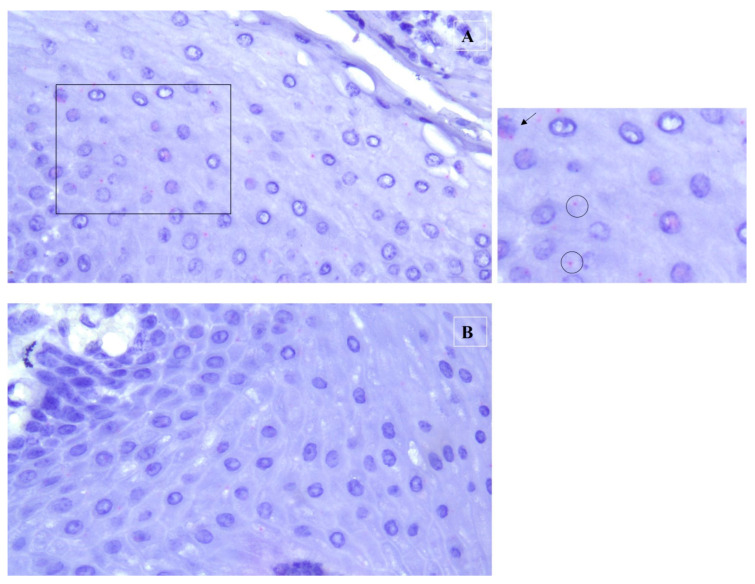
RNAscope assay. (**A**) Tissue sections hybridized with the probe targeting BPV-1 E5-E6-E7 mRNA (V-BPV-E). A close-up view of the inset is visible in the right panel. Specific staining was observed in the congenital papilloma under investigation. In detail, scattered red dots were detected within the cytoplasms (empty circles) and nuclei (black arrow) of epithelial cells. (**B**) On the contrary, no specific staining was observed in the negative control. Final magnification: ×400.

After this test, to evaluate the mRNA localization, RNA in situ hybridization was performed using the RNAscope kit (Advanced Cell Diagnostics Inc., Hayward, CA, USA), according to the manufacturer’s guidelines, as described in [9]. The RNAscope probe used was the V-BPV-E-bovine-papillomavirus-1 complete genome E5 E6 E7 (Catalog Number 416831) and was designed to detect the mRNA expression of the E5, E6, and E7 genes (National Center for Biotechnology Information Reference Sequence NC_001522.1). FFPE tissue sections (of 5 μm thickness) were deparaffinized in xylene, and were subsequently dehydrated in 100% ethanol. Tissue sections were exposed to hydrogen peroxide for 10 min, followed by incubation in a target retrieval reagent maintained at a boiling temperature (98 to 102 °C) using a hot plate for 15 min, rinsed in deionized water, and immediately treated with Protease Plus at 40 °C for 30 min in a hybridization oven (TopBrite- Resnova, RM, Italy). The tissue sections were then incubated at 40 °C with a target probe of BPV-1 for 2 h. The target–probe hybridization was followed by a series of target-specific signal-amplification steps. After each hybridization step, slides were washed with wash buffer at room temperature twice, followed by staining with Fast Red dye. The sections were then counterstained with 50% hematoxylin staining solution, and dehydrated using xylene, before being mounted with a mounting medium (Eukitt). The positive signals were present in the form of punctate cytoplasmic and nuclear red staining that was higher than the signal on the negative control slide. Assays using FFPE specimens were performed in parallel with positive and negative control probes (positive probe: Ec-PPIB; negative control probe: DapB), to ensure interpretable results (Figure 5).

## 3. Discussion

Papillomaviruses are regarded as very important oncogenic viruses, able to induce benign and often self-limiting epithelial neoplasms, which can occasionally progress to malignancy [10,11]. In humans, cervical cancer represents the most common PV-associated cancer [12]. Moreover, the role of human PVs as causative agents of anogenital, head, and neck cancers is widely accepted [13]. Likewise, PVs have been associated with malignant neoplasms in several animal species [11]. In horses, a growing body of evidence suggests that EcPV2 infection contributes to the etiopathogenesis of squamous cell carcinomas, which can affect the external genitalia, and laryngeal and gastric mucosa [14,15]. Other types of equine papillomaviruses have been associated with different lesions. In particular, papillomas seem to be caused by EcPV1, EcPV3-6, and EcPV8, while EcPV9 and EcPV10, to date, are not associated with specific pathology [16,17,18].

Papillomaviruses are strictly species-specific and show a distinct tropism towards epithelial cells. Delta-PVs (e.g., BPV1 and BPV2) are exceptions to this general rule, as they have a wider host range and can infect different cell types [11,19]. A recent case report by Savini and co-workers (2020) demonstrated the ability of BPV1 to cause proliferative lesions in sheep. These results suggest a different pathogenetic role of BPV-1, and confirm the ability of BPV1 to infect different species, also causing a non-sarcoid outcome on cutaneous surfaces [20]. Notably, phylogenetic investigations revealed that BPV1 originated in cattle, while multiple cross-species transmissions occurred into horses [21]. The causal association of BPV1 and BPV2 infection with equid sarcoid has been unequivocally demonstrated [22]. It was assumed that BPV1/2 infections were strictly confined to dermal fibroblasts in the horses, wherein they reside in a non-productive episomal form. Consequently, horses have been long considered a dead-end host for BPV1/2 [14]. However, BPV1 infection has been demonstrated to involve the epidermis of the horse, where it may be productive, albeit at low level [23]. In addition, BPV1 can early-infect PBMCs in young horses, likely inducing a viremic phase [24], and the BPV1 genome has been detected in the placenta and blood of newborn foals [25].

Overall, biomolecular investigations clearly demonstrated BPV1 infection in the equine congenital papilloma under study. As a matter of fact, the detection of BPV1 oncogene mRNA and its cytoplasmatic localization ruled out any incidental contamination at the time of sampling and/or during laboratory tests. Moreover, according to the literature, such data would be unlikely to result from the selective infection of PBMCs, considering the high viral load detected (the BPV1 DNA was 100-fold more concentrated than the equine genome, when compared with the β2M), as well as the transcription of the L1 gene [24,26,27]. The present case report is apparently in contrast with those currently available in the literature [6,7]. However, we remark that White et al. [6] and Postey et al. [7] only ruled out the presence of EcPVs in equine congenital papillomas, while no data have ever been provided about BPV1 infection in such lesions.

Although PV infections mainly spread through horizontal transmission, a growing interest exists around intrauterine and perinatal transmission. In humans, the PV (human PV, HPV) genome has been detected in the amniotic fluid, placenta, and umbilical cord [28,29], and transplacental infection might occur through the hematogenous or ascending route, from the maternal genital tract [30]. The hypothesis of vertical transmission has been further corroborated in cattle, as BPV2 was shown to productively infect the uterine epithelium and chorionic placenta [31,32]. Accordingly, some evidence supports BPV vertical transmission in sheep [33]. The mechanism of BPV transmission in horses is still debated; the BPV genome has been detected in the equine placenta, and intrauterine transmission has been speculated in this animal species [25]. Moreover, Silva and co-workers demonstrated the virus’ presence in the PBMC and semen of healthy horses [27,34]. Therefore, recently, it was speculated that, as in humans, infection could be ascribable to oocyte fecundation [35].

Considering this, the detection of BPV1 in congenital papillomas argues in favor of its transplacental transmission in horses, stimulating further investigation to identify the risk factors, prevalence, and clinical relevance of such event.

Congenital papillomas have been repeatedly reported in animals (including horses) and regarded as hamartomatous lesions, with no viral origin having been demonstrated [1,6,7]. It is worth noting that Roperto et al. [33] recently reported BPV-associated congenital lesions in lambs after transplacental infection. Of course, the conclusive demonstration of the viral etiology (if any) of equine congenital papilloma goes well beyond the scope of the present case report. Moreover, the molecular detection of BPV1 in newborn healthy foals [25], along with the conflicting data about BPV1 infection in the skin of healthy horses [36,37], add further concerns about the significance of BPV1 in equine congenital papilloma. In our opinion, the case described herein suggests that BPV vertical transmission might have clinical relevance in different animal species, further highlighting the biological plasticity of delta-PVs. As a matter of fact, BPV1/2 have been shown to infect several animal species, targeting a wide range of cell types and causing different neoplastic lesions depending on the host features (animal species, age, route of transmission).

## 4. Conclusions

The present case report found BPV1 infection in an equine congenital papilloma, with very high viral load. This stimulates further investigations, even on archived samples, aiming to clarify the etiology of equine congenital papilloma and the clinical relevance, if any, of BPV1 vertical transmission in horses.

## Figures and Tables

**Table 1:** Primer set and probes used in the virological investigations.

Target Gene	Primer Sequences	Amplicon Length	Accession Number
EcPV2-L1	F-5′-TTGTCCAGGAGAGGGGTTAG-3′	80	NC_012123.1
	R-5′-TGCCTTCCTTTTCTTGGTGG-3′		
p-EcPV2-L1	FAM-CGTCCAGCACCTTCGACCACCA-TAMRA		
EcPV9-L1	F-5′-TTC ATC CCA GCT TGA GAC CA-3′	116	MN117918.1
	R-5′-GCA GAT CAA TGG TCC AGA AGG-3′		
p-EcPV9-L1	p-FAM-ATT GCC TCC TCA GCC ACC CG-TAMRA		
EcPV10-L1	F-5′-GTG TCA CAG GTA ACC CCC TG-3′	174	OP870083
	R-5′-AAG CGT GTC TTC CTC CAG TG-3′		
p-EcPV10-L1	p-FAM-TGC TGG TGG GTT GCA AGC CC-TAMRA		
BPV1-L1	F-5′-CAG GAC TGT TCA CAA CCC AA-3′	96	NC_001522.1
	R-5′-CCC AGT TAC AGT ACC TCC AA-3′		
p-BPV1-L1	p-FAM-TGC AGG TGT CCA GAG GGC AG-TAMRA		
BPV2-L1	F-5′-ACA GCC CGT CCA TGT GTT A-3′	115	MF045490
	R-5′-TCA GCA GCA CCA AAC CCT AT-3′		
p-BPV2-L1	p-FAM-AGA AAA TGG TGC GTG TCC TCC T-TAMRA		
BPV1-E5	F-5′-TGCTTCAATGCAACTGCTGCT-3′	77	MZ310894
	R-5′-AGGAGCACTCAAAATGATCCCAG-3′		
p-BPV1-E5	p-FAM-ACTCTTGTTTTTTCTTGTA-TAMRA		
BPV1-E6	F-5′-TGCTACTGTGGGGGCAAACT-3′	110	MZ310894
	R-5′-CAGTCGTAGCAGCGTCCTCT-3′		
p-BPV1-E6	p-FAM-AGCCTTTCTGCAAAACCAGAGCT-TAMRA		
BPV1-E7	F-5′-GCTGTGGAAACTGCGGAAAA-3′	122	MZ310894
	R-5′-GCGAGATTCACAACGTGGAC-3′		
p-BPV1-E7	p-FAM-GCTGACTTTTGCAGTGAAGACCAGC-TAMRA		
BPV13-L1	F-5′-GCA CCC CAC TTT TAA TGC CT-3′	87	NC_030795
	R-5′-TCC TGT TTG CTT CCT GTC ATC-3′		
p-BPV13-L1	p-FAM-AGG AAA GTG ACC AGC CAA ACA ACA-TAMRA		
*B2M (Beta2-microglobulin)*	F-5′-GGCTACTCTCCCTGACTGG-3′	135	NM_001082502.3
	R-5′-TCAATCTCAGGCGGATGGAA-3′		
p-*B2M*	p-FAM-ACTCACGTCACCCAGCAGAGA-TAMRA		

**Table 2:** Real-time PCR targeting the PV genome. Data are expressed as Cq mean value ± standard deviation.

Sample	Real-Time PCR					
	B2M	EcPV2-L1	EcPV9-L1	EcPV10-L1	BPV2-L1	BPV13-L1
	29.4 ± 0.1	>38	>38	>38	>38	>38
	**B2M**	**BPV1-E5**	**BPV1-E6**	**BPV1-E7**	**BPV1-L1**	
	29.1 ± 0.1	21.5 ± 0.2	21.7 ± 0.4	21.5 ± 0.2	22.5 ± 0.5	

**Table 3:** BPV1 oncogene expression. Data are expressed as Cq mean value ± standard deviation.

Sample	Real-Time PCR				
	B2M	BPV1-E5	BPV1-E6	BPV1-E7	BPV1-L1
	31.4 ± 0.2	30.1 ± 0.2	34.2 ± 0.2	21.5 ± 0.2	37.1 ± 0.3

## Data Availability

All data deriving from the study are given in the article text.

## Supplementary Materials

The following supporting information can be downloaded at: https://www.mdpi.com/article/10.3390/pathogens12081059/s1, Figure S1: Curve of Real Time PCR to detection of BPV1.
