# Supplementary material for: Bovine Papillomavirus Type 1 Infection in an Equine Congenital Papilloma
Source: Pathogens. 2023 Aug 18;12(8):1059. doi: 10.3390/pathogens12081059 (PMC10458069; doi:10.3390/pathogens12081059)
Supplement: Supplementary file 1 [file pathogens-12-01059-s001.zip › pathogens-2377915-supplementary.pdf]

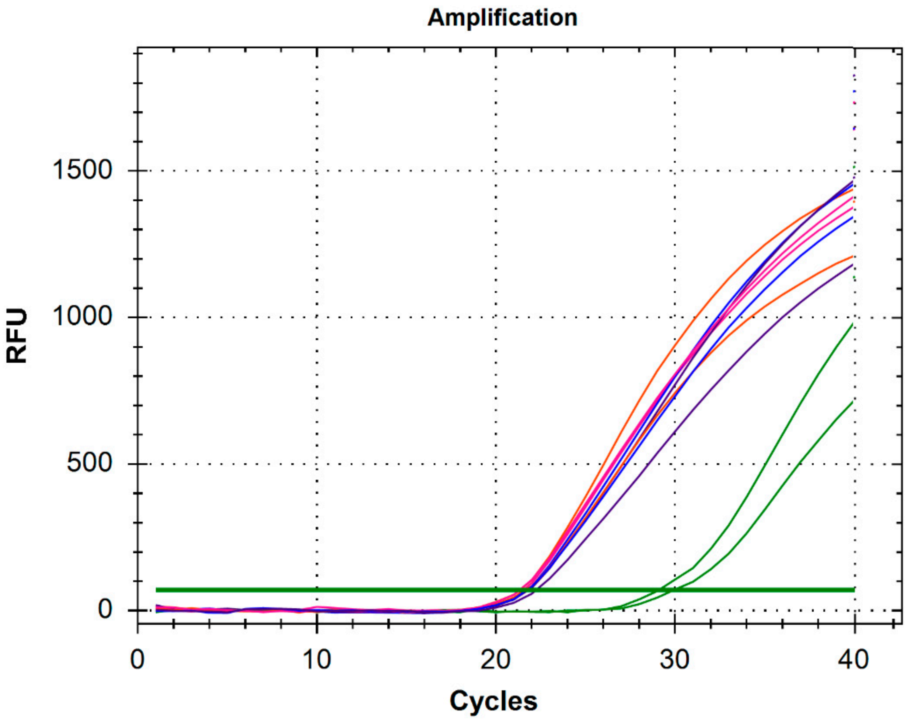

Figure S1: Curve of Real Time PCR to detection of BPV1

Different gene are showed by differents coulors:

Violet two replicats of L1

Green two replicates of B2M

Orange two replicates of E5

Blu two replicates of E6

Fuxia two replicates of E7
